# Supplementary material for: Protective activities of distinct omega-3 enriched oils are linked to their ability to upregulate specialized pro-resolving mediators
Source: PLoS One. 2020 Dec 16;15(12):e0242543. doi: 10.1371/journal.pone.0242543 (PMC7743945; doi:10.1371/journal.pone.0242543)
Supplement: S1 File — (DOCX) [file pone.0242543.s001.docx]

**Protective Activities Of Distinct Omega-3 Enriched Oils Are Linked To Their Ability To Upregulate Specialized Pro-Resolving Mediators**

Agua Sobrino ^1^, Mary E. Walker ^1^, Romain A. Colas ^1^, Jesmond Dalli^1,2^

*Affiliations*

^1^ William Harvey Research Institute, Barts and The London School of Medicine and Dentistry, Queen Mary University of London, Charterhouse Square, London, EC1M 6BQ, UK.

^2^ Centre for Inflammation and Therapeutic Innovation, Queen Mary University of London, London, UK.

Corresponding author: Prof Jesmond Dalli Ph.D, Lipid Mediator Unit, William Harvey Research Institute, John Vane Science Centre, Charterhouse Square, London EC1M 6BQ. E-mail: [j.dalli@qmul.ac.uk](mailto:j.dalli@qmul.ac.uk) Tel: +44 (0) 207 882 8263

**Supplemental Table 1. Regulation of macrophage phenotypic markers by omega-3 enriched oils.**

|  | **M0 Macrophages** | | | **+LPS** | | | **+LPS**  **+Algal Oil** | | | **+LPS**  **+Meganol D** | | | **+LPS**  **+Meganol E** | | | **+LPS**  **+Meganol ED** | | |
| --- | --- | --- | --- | --- | --- | --- | --- | --- | --- | --- | --- | --- | --- | --- | --- | --- | --- | --- |
| **Macrophage Receptors** | Mean |  | SEM | Mean |  | SEM | Mean |  | SEM | Mean |  | SEM | Mean |  | SEM | Mean |  | SEM |
| CD68 | 185.9 | ± | 16.3 | 200.0 | ± | 13.7 | 244.5 | ± | 72.5 | 256.0 | ± | 38.9 | 230.8 | ± | 18.6 | 213.3 | ± | 10.8 |
| CD206 | 46.7 | ± | 6.9 | 43.8 | ± | 5.4 | 29.8 | ± | 3.6 | 43.0 | ± | 6.1 | 40.1 | ± | 7.8 | 36.6 | ± | 6.6 |
| CD36 | 659.7 | ± | 256.4 | 468.6 | ± | 44.5 | 568.5 | ± | 90.2 | 477.8 | ± | 85.8 | 609.3 | ± | 106.2 | 904.0 | ± | 130.1 |
| CD14 | 287.6 | ± | 87.3 | 350.1 | ± | 32.9 | 479.0 | ± | 98.2 | 384.5 | ± | 83.7 | 425.3 | ± | 110.1 | 525.0 | ± | 146.3 |
| MerTK | 169.4 | ± | 7.7 | 192.6 | ± | 40.7 | 144.8 | ± | 18.1 | 239.0 | ± | 47.0 | 177.8 | ± | 24.9 | 180.3 | ± | 26.5 |
| CD80 | 186.6 | ± | 9.9 | 216.5 | ± | 23.3 | 142.5 | ± | 28.7 | 204.8 | ± | 47.6 | 188.5 | ± | 35.8 | 209.3 | ± | 45.4 |
| CD300f | 133.7 | ± | 42.2 | 176.9 | ± | 24.2 | 167.4 | ± | 73.0 | 176.8 | ± | 58.2 | 163.1 | ± | 44.0 | 208.8 | ± | 67.2 |
| CD163 | 137.0 | ± | 45.00 | 178.0 | ± | 28.9 | 146.9 | ± | 55.4 | 170.7 | ± | 69.2 | 157.7 | ± | 52.5 | 210.3 | ± | 70.7 |

Human monocyte-derived macrophages were incubated with vehicle (PBS) or LPS (1 ng/ mL, 24 h) with or without the indicated oils (200 pg of SPM precursors, 24 h; *see methods for details*). The expression of phenotypic markers was assessed using flow cytometry. Results are mean n = 4 donors. Expression of lineage markers is expressed as mean fluorescence intensity (MFI) units.

**Supplementary Table 2: Differential lipid mediator profiles in human monocyte derived macrophages incubated with omega-3 enriched oils.**

|  | **Vehicle** | | | **Algal Oil** | | | **Meganol D** | | | **Meganol E** | | | **Meganol ED** | | |
| --- | --- | --- | --- | --- | --- | --- | --- | --- | --- | --- | --- | --- | --- | --- | --- |
| **DHA bioactive metabolome** | Mean |  | SEM | Mean |  | SEM | Mean |  | SEM | Mean |  | SEM | Mean |  | SEM |
| RvD1 | 1.8 | ± | 1.7 | 343.3 | ± | 185.4 | 14,154.5 | ± | 11,384.6 | 7,920.2 | ± | 3,471.0 | 320.2 | ± | 189.9 |
| RvD2 | 0.0 | ± | 0.0 | 18.2 | ± | 15.8 | 3,118.0 | ± | 2,805.6 | 2,751.2 | ± | 2,046.2 | 173.5 | ± | 163.6 |
| RvD3 | 6.6 | ± | 2.1 | 8.2 | ± | 6.7 | 113.2 | ± | 98.3 | 106.0 | ± | 72.8 | 8.5 | ± | 4.0 |
| RvD4 | 7.3 | ± | 4.0 | 14.4 | ± | 9.6 | 130.0 | ± | 107.1 | 11.1 | ± | 11.3 | 4.4 | ± | 2.8 |
| RvD5 | 21.0 | ± | 9.8 | 38.3 | ± | 25.7 | 249.1 | ± | 190.7 | 247.7 | ± | 138.8 | 28.1 | ± | 12.7 |
| RvD6 | 6.4 | ± | 3.9 | 9.6 | ± | 4.0 | 143.6 | ± | 113.9 | 205.6 | ± | 73.6 | 9.0 | ± | 6.2 |
| 17R-RvD1 | 0.8 | ± | 0.8 | 120.5 | ± | 167.1 | 297.9 | ± | 380.6 | 653.7 | ± | 578.0 | 87.1 | ± | 44.6 |
| 17R-RvD3 | 1.0 | ± | 0.7 | 12.4 | ± | 3.3 | 1,175.5 | ± | 1,002.6 | 651.2 | ± | 427.1 | 16.7 | ± | 15.0 |
| PD1 | 1.8 | ± | 1.4 | 3.5 | ± | 1.4 | 72.8 | ± | 58.8 | 21.9 | ± | 16.0 | 4.0 | ± | 2.5 |
| 17R-PD1 | 2.5 | ± | 1.6 | 20.7 | ± | 15.6 | 406.8 | ± | 337.5 | 253.4 | ± | 203.9 | 26.7 | ± | 19.8 |
| 10S.17S-diHDHA | 10.3 | ± | 6.1 | 51.7 | ± | 37.9 | 95.0 | ± | 28.0 | 160.8 | ± | 120.7 | 27.8 | ± | 12.5 |
| 22-OH-PD1 | 0.0 | ± | 0.0 | 0.6 | ± | 1.0 | 25.1 | ± | 44.3 | 83.2 | ± | 111.1 | 1.2 | ± | 2.1 |
| MaR1 | 9.2 | ± | 7.1 | 96.1 | ± | 49.4 | 1,058.3 | ± | 1,355.6 | 812.4 | ± | 786.9 | 102.5 | ± | 73.5 |
| MaR2 | 14.0 | ± | 8.3 | 83.8 | ± | 21.1 | 2,569.6 | ± | 1,881.1 | 2,300.4 | ± | 1148.9 | 68.7 | ± | 50.4 |
| 7S.14S-diHDHA | 21.2 | ± | 15.7 | 86.9 | ± | 55.8 | 443.7 | ± | 273.8 | 543.1 | ± | 473.5 | 51.9 | ± | 19.2 |
| 22-OH-MaR1 | 95.0 | ± | 90.7 | 146.6 | ± | 132.3 | 988.0 | ± | 1019.6 | 356.6 | ± | 323.8 | 70.8 | ± | 34.2 |
| 14-oxo-MaR1 | 1.0 | ± | 1.7 | 0.0 | ± | 0.0 | 40.5 | ± | 39.2 | 29.1 | ± | 29.8 | 0.7 | ± | 1.2 |
| 4S.14S-diHDHA | 6.5 | ± | 4.4 | 9.8 | ± | 2.8 | 276.2 | ± | 203.4 | 268.7 | ± | 67.7 | 22.2 | ± | 14.8 |
| **n-3 DPA bioactive metabolome** |  |  |  |  |  |  |  |  |  |  |  |  |  |  |  |
| RvT1 | 3.2 | ± | 0.9 | 647.1 | ± | 573.6 | 5,166.1 | ± | 2,131.1 | 1472.7 | ± | 817.6 | 321.8 | ± | 124.5 |
| RvT2 | 4.1 | ± | 3.7 | 35.9 | ± | 32.1 | 504.8 | ± | 349.0 | 198.3 | ± | 101.6 | 26.9 | ± | 12.1 |
| RvT3 | 0.8 | ± | 0.6 | 10.9 | ± | 8.0 | 175.3 | ± | 91.6 | 67.1 | ± | 24.9 | 3.0 | ± | 2.3 |
| RvT4 | 27.7 | ± | 26.8 | 53.8 | ± | 20.5 | 3,743.9 | ± | 3,254.5 | 3,118.0 | ± | 2,406.3 | 66.5 | ± | 28.9 |
| RvD1_n-3 DPA_ | 1.9 | ± | 1.0 | 3.3 | ± | 3.9 | 481.0 | ± | 403.8 | 195.6 | ± | 110.0 | 7.6 | ± | 5.3 |
| RvD2 _n-3 DPA_ |  | - |  | 1.9 | ± | 2.7 | 144.6 | ± | 245.4 | 3.1 | ± | 4.3 | 145.1 | ± | 190.8 |
| RvD5 _n-3 DPA_ | 4.7 | ± | 1.2 | 3.7 | ± | 1.0 | 94.9 | ± | 64.2 | 90.3 | ± | 65.4 | 2.5 | ± | 2.7 |
| PD1_n-3 DPA_ | 1.5 | ± | 1.5 | 1.9 | ± | 0.7 | 130.3 | ± | 142.3 | 109.8 | ± | 85.3 | 1.9 | ± | 0.5 |
| PD2_n-3 DPA_ | 0.1 | ± | 0.2 |  | - |  | 5.8 | ± | 4.0 | 11.9 | ± | 4.0 | 0.5 | ± | 0.9 |
| 10S. 17S-diHDPA | 1.3 | ± | 0.5 | 2.1 | ± | 1.4 | 77.2 | ± | 59.5 | 65.0 | ± | 53.1 | 2.1 | ± | 1.4 |
| MaR1_n-3 DPA_ | 0.8 | ± | 1.0 | 0.6 | ± | 1.0 | 18.9 | ± | 19.9 | 39.9 | ± | 36.5 | 2.2 | ± | 2.4 |
| MaR2_n-3 DPA_ | 14.8 | ± | 9.0 | 25.2 | ± | 16.6 | 289.4 | ± | 250.5 | 236.1 | ± | 185.4 | 14.0 | ± | 5.7 |
| 7S.14S-diHDPA | 2.8 | ± | 2.2 | 1.5 | ± | 1.5 | 19.1 | ± | 14.5 | 35.4 | ± | 20.3 | 1.8 | ± | 1.8 |
| **EPA bioactive metabolome** |  |  |  |  |  |  |  |  |  |  |  |  |  |  |  |
| RvE1 | 0.4 | ± | 0.7 | 1.0 | ± | 1.7 | 11.4 | ± | 12.1 |  | - |  | 4.3 | ± | 4.4 |
| RvE2 | 16.6 | ± | 7.7 | 30.5 | ± | 18.0 | 1,118.8 | ± | 680.1 | 449.4 | ± | 153.6 | 117.3 | ± | 56.1 |
| RvE3 | 4.0 | ± | 4.3 | 48.1 | ± | 35.5 | 1,471.3 | ± | 1,638.5 | 660.2 | ± | 513.2 | 112.0 | ± | 155.3 |
| **AA bioactive metabolome** |  |  |  |  |  |  |  |  |  |  |  |  |  |  |  |
| LXA_4_ | 17.2 | ± | 7.3 | 12.0 | ± | 2.7 | 114.4 | ± | 35.2 | 22.3 | ± | 13.2 | 63.4 | ± | 25.2 |
| LXB_4_ | 291.6 | ± | 331.5 | 921.0 | ± | 968.7 | 12,163.1 | ± | 7,953.0 | 3,353.5 | ± | 1,126.3 | 3,359.4 | ± | 1,224.6 |
| 5S.15S-diHETE | 45.1 | ± | 32.0 | 53.0 | ± | 32.4 | 96.4 | ± | 21.8 | 50.9 | ± | 14.1 | 62.0 | ± | 35.2 |
| 13.14-dehydro-15-oxo-LXA_4_ | 53.8 | ± | 18.2 | 154.6 | ± | 66.9 | 2,555.3 | ± | 1,794.4 | 2,041.3 | ± | 1,283.4 | 575.2 | ± | 297.3 |
| 15-oxo-LXA_4_ | 1.0 | ± | 1.2 | 3.2 | ± | 0.9 | 45.1 | ± | 32.2 | 30.7 | ± | 11.6 | 20.7 | ± | 16.1 |
| 15-epi-LXA_4_ | 129.0 | ± | 61.5 | 104.5 | ± | 53.6 | 304.1 | ± | 89.6 | 177.4 | ± | 31.7 | 98.3 | ± | 62.9 |
| 15-epi-LXB_4_ | 39.7 | ± | 35.2 | 61.1 | ± | 50.0 | 152.9 | ± | 63.8 | 78.7 | ± | 42.2 | 90.1 | ± | 98.1 |
| LTB_4_ | 21.5 | ± | 20.7 | 19.2 | ± | 20.8 | 104.7 | ± | 58.9 | 74.2 | ± | 27.1 | 29.0 | ± | 19.7 |
| 5S.12S-diHETE | 63.7 | ± | 26.9 | 56.0 | ± | 32.1 | 118.2 | ± | 37.5 | 93.7 | ± | 45.0 | 58.4 | ± | 18.8 |
| 20-OH-LTB_4_ |  | - |  |  | - |  | 13.8 | ± | 24.5 | 1.5 | ± | 2.6 |  | - |  |
| PGE_2_ | 109.4 | ± | 86.4 | 49.1 | ± | 18.0 | 98.5 | ± | 34.6 | 117.7 | ± | 36.4 | 76.4 | ± | 24.9 |
| PGD_2_ | 46.2 | ± | 45.8 | 45.6 | ± | 28.2 | 336.1 | ± | 229.0 | 216.3 | ± | 107.9 | 111.7 | ± | 55.7 |
| PGF_2a_ | 75.6 | ± | 26.6 | 74.4 | ± | 49.3 | 141.9 | ± | 109.8 | 189.5 | ± | 163.8 | 127.2 | ± | 85.3 |
| TxB_2_ | 456.1 | ± | 350.1 | 518.2 | ± | 461.1 | 268.4 | ± | 221.3 | 2,527.9 | ± | 2,771.1 | 1,389.0 | ± | 1,326.3 |

Human monocyte-derived macrophages were incubated with LPS (1 ng/ mL, 24 h) and with the indicated oils (normalized to 200 pg of SPM precursors, 24h) or vehicle (PBS + 0.1 % EtOH). Lipid mediator profiles were assessed using LC-MS/MS-based profiling. Results are mean n = 4 donors. Concentration is expressed as pg / incubation.

**Supplementary Table 3. Algal Oil and Meganol D upregulate plasma SPM concentrations in APOE^-/-^ fed a Western Diet.**

|  | **Vehicle** | | | **Algal Oil** | | | | **Meganol D** | | | | |
| --- | --- | --- | --- | --- | --- | --- | --- | --- | --- | --- | --- | --- |
| **DHA bioactive metabolome** | Mean |  | SEM | Mean |  | | SEM | Mean | |  | | SEM |
| RvD1 | 8.0 | ± | 3.9 | 21.2 | ± | | 4.9 | 23.1 | | ± | | 7.6 |
| RvD2 | 2.6 | ± | 1.3 | 4.1 | ± | | 2.8 | 5.1 | | ± | | 4.5 |
| RvD3 | 2.0 | ± | 1.1 | 0.8 | ± | | 0.8 | 2.5 | | ± | | 1.8 |
| RvD4 | 15.3 | ± | 3.4 | 15.9 | ± | | 16.0 | 17.7 | | ± | | 4.3 |
| RvD5 | 3.5 | ± | 1.4 | 12.8 | ± | | 6.5 | 20.8 | | ± | | 12.8 |
| RvD6 | 2.9 | ± | 1.5 | 5.3 | ± | | 3.0 | 2.6 | | ± | | 0.8 |
| 17R-RvD1 | 5.1 | ± | 2.8 | 3.3 | ± | | 1.8 | 5.9 | | ± | | 3.3 |
| 17R-RvD3 | 1.0 | ± | 1.3 | 1.2 | ± | | 1.0 | 1.3 | | ± | | 1.6 |
| PD1 | 3.8 | ± | 1.5 | 4.8 | ± | | 2.6 | 22.9 | | ± | | 34.4 |
| 17R-PD1 | 36.9 | ± | 8.4 | 46.6 | ± | | 13.7 | 57.1 | | ± | | 28.5 |
| 10S.17S-diHDHA | 0.0 | ± | 0.0 | 0.0 | ± | | 0.0 | 1.9 | | ± | | 2.2 |
| 22-OH-PD1 | 2.2 | ± | 2.3 | 1.6 | ± | | 1.4 | 7.4 | | ± | | 4.0 |
| MaR1 | 255.5 | ± | 84.5 | 271.8 | ± | | 209.6 | 322.8 | | ± | | 123.4 |
| MaR2 | 15.5 | ± | 10.0 | 24.0 | ± | | 6.7 | 55.8 | | ± | | 29.8 |
| 22-OH-MaR1 | 175.3 | ± | 87.0 | 242.5 | ± | | 103.8 | 274.8 | | ± | | 73.2 |
| 14-oxo-MaR1 | 0.0 | ± | 0.0 | 0.4 | ± | | 0.6 | 0.0 | | ± | | 0.0 |
| 7S.14S-diHDHA | 0.5 | ± | 0.8 | 14.7 | ± | | 14.1 | 14.7 | | ± | | 9.8 |
| 4S.14S-diHDHA | 58.1 | ± | 43.9 | 52.9 | ± | | 14.0 | 111.9 | | ± | | 54.2 |
| **n-3 DPA bioactive metabolome** |  |  |  |  |  | |  |  | |  | |  |
| RvT1 | 10.2 | ± | 2.5 | 44.5 | ± | 13.1 | | 18.9 | ± | | 2.0 | |
| RvT2 | 3.1 | ± | 4.3 | 1.2 | ± | 1.3 | | 0.6 | ± | | 0.7 | |
| RvT3 | 6.1 | ± | 3.3 | 14.0 | ± | 7.9 | | 8.3 | ± | | 4.7 | |
| RvT4 | 2.5 | ± | 1.8 | 9.0 | ± | 7.7 | | 2.3 | ± | | 1.2 | |
| RvD1_n-3 DPA_ | 3.7 | ± | 1.9 | 6.9 | ± | 7.1 | | 1.4 | ± | | 1.4 | |
| RvD2_n-3 DPA_ | 1.1 | ± | 1.1 | 0.6 | ± | 0.5 | | 1.2 | ± | | 1.1 | |
| RvD5_n-3 DPA_ | 2.9 | ± | 3.1 | 2.9 | ± | 3.1 | | 5.8 | ± | | 4.2 | |
| PD1_n-3 DPA_ | 0.9 | ± | 0.6 | 0.0 | ± | 0.0 | | 0.5 | ± | | 0.7 | |
| PD2_n-3 DPA_ | 0.9 | ± | 0.9 | 3.0 | ± | 1.2 | | 6.5 | ± | | 0.9 | |
| 10S. 17S-diHDPA | 1.4 | ± | 1.1 | 0.4 | ± | 0.3 | | 1.2 | ± | | 1.0 | |
| 22-OH-PD1n-3 DPA | 0.0 | ± | 0.0 | 0.2 | ± | 0.3 | | 0.0 | ± | | 0.0 | |
| MaR1_n-3 DPA_ | 1.4 | ± | 1.5 | 4.5 | ± | 2.3 | | 5.4 | ± | | 3.3 | |
| MaR2_n-3 DPA_ | 0.0 | ± | 0.0 | 0.0 | ± | 0.0 | | 0.0 | ± | | 0.0 | |
| 7S.14S-diHDPA | 4.6 | ± | 1.6 | 7.6 | ± | 7.1 | | 7.9 | ± | | 4.0 | |
| **EPA bioactive metabolome** |  |  |  |  |  |  | |  |  | |  | |
| RvE1 | 8.8 | ± | 8.9 | 2.8 | ± | 2.1 | | 9.4 | ± | | 8.9 | |
| RvE2 | 6.9 | ± | 8.3 | 0.0 | ± | 0.0 | | 5.6 | ± | | 10.6 | |
| RvE3 | 96.1 | ± | 37.1 | 368.4 | ± | 230.0 | | 347.6 | ± | | 369.0 | |
| **AA bioactive metabolome** |  |  |  |  |  |  | |  |  | |  | |
| LXA_4_ | 0.5 | ± | 0.9 | 8.9 | ± | 11.1 | | 4.2 | ± | | 4.3 | |
| LXB_4_ | 22,176.8 | ± | 5,719.3 | 30,163.8 | ± | 22,320.1 | | 21,295.7 | ± | | 2,551.4 | |
| 5S.15S-diHETE | 38.7 | ± | 22.9 | 12.3 | ± | 6.4 | | 41.7 | ± | | 27.6 | |
| 13.14-dehydro-15-oxo-LXA_4_ | 182.4 | ± | 163.3 | 88.4 | ± | 35.0 | | 193.6 | ± | | 234.7 | |
| 15-oxo-LXA_4_ | 6,246.8 | ± | 584.3 | 6,715.5 | ± | 3156.3 | | 6,631.1 | ± | | 950.6 | |
| 15-epi-LXA_4_ | 225.2 | ± | 94.8 | 174.0 | ± | 128.8 | | 245.5 | ± | | 76.5 | |
| 15-epi-LXB_4_ | 9.2 | ± | 5.0 | 3.9 | ± | 1.6 | | 12.8 | ± | | 7.8 | |
| LTB_4_ | 1,485.5 | ± | 1,554.1 | 1,182.2 | ± | 899.6 | | 2,125.6 | ± | | 2,065.0 | |
| 5S.12S-diHETE | 231.3 | ± | 199.6 | 104.7 | ± | 86.7 | | 372.8 | ± | | 276.3 | |
| 20-OH-LTB_4_ | 169.8 | ± | 54.6 | 174.0 | ± | 91.0 | | 154.0 | ± | | 39.4 | |
| PGE_2_ | 215.4 | ± | 98.5 | 203.8 | ± | 75.2 | | 172.8 | ± | | 43.7 | |
| PGD_2_ | 71.6 | ± | 35.5 | 81.0 | ± | 34.6 | | 78.5 | ± | | 22.7 | |
| PGF_2a_ | 899.1 | ± | 530.3 | 1,571.9 | ± | 1,586.6 | | 1,516.2 | ± | | 923.5 | |
| TxB_2_ | 10.2 | ± | 2.5 | 44.5 | ± | 13.1 | | 18.9 | ± | | 2.0 | |

Mice were fed a western diet for 5 weeks, then they were administered Algal oil or Meganol D at dose of 9.2 pg of SPM precursors per mouse/day or vehicle (0.1 % EtOH) for a 2-week period. Blood was collected and plasma lipid mediator concentrations were determined using LC-MS/MS based lipid mediator profiling. Results are mean n = 5 mice (Vehicle and Algal Oil) and n = 6 mice (Meganol D). Concentration is expressed as pg /200µl of plasma.
